# A Case of Type 2 Youssef's Syndrome following Caesarean Section for Placenta Previa Totalis

**DOI:** 10.1155/2016/4505467

**Published:** 2016-10-10

**Authors:** Sefa Kurt, Funda Obuz

**Affiliations:** ^1^Department of Obstetrics and Gynecology, Dokuz Eylül University Faculty of Medicine, İzmir, Turkey; ^2^Department of Radiology, Dokuz Eylül University Faculty of Medicine, İzmir, Turkey

## Abstract

Vesicouterine fistula is a rare type of urogenital fistulas. It is most commonly observed after cesarean section (C/S) due to iatrogenic reasons. In this article, a case of a vesicouterine fistula which developed after C/S operation is presented. This was the patient's second C/S and this time placenta previa totalis was the primary pathology. Since it is a rare complication, we found it interesting, and, in this article, this clinical problem was discussed with details about diagnosis and treatment in light of the literature.

## 1. Introduction

Vesicouterine fistulas (VUFs) are classified in the group of urogenital fistulas (UGFs). These rare fistulas make up 1–4% of all UGFs [1]. Previously, VUFs were regarded as a complication of extended labor and difficult delivery; today, they are mostly observed as a complication after caesarean delivery or certain gynecological operations.* The VUFs, which are observed after caesarean delivery, are not accompanied by urinary incontinence, are characterized by amenorrhea and cyclic haematuria, and are called Youssef's syndrome* [2]. In this article, a VUF case that developed after the second caesarean due to placenta previa totalis was presented.

## 2. Case Presentation

**Figure 1:**
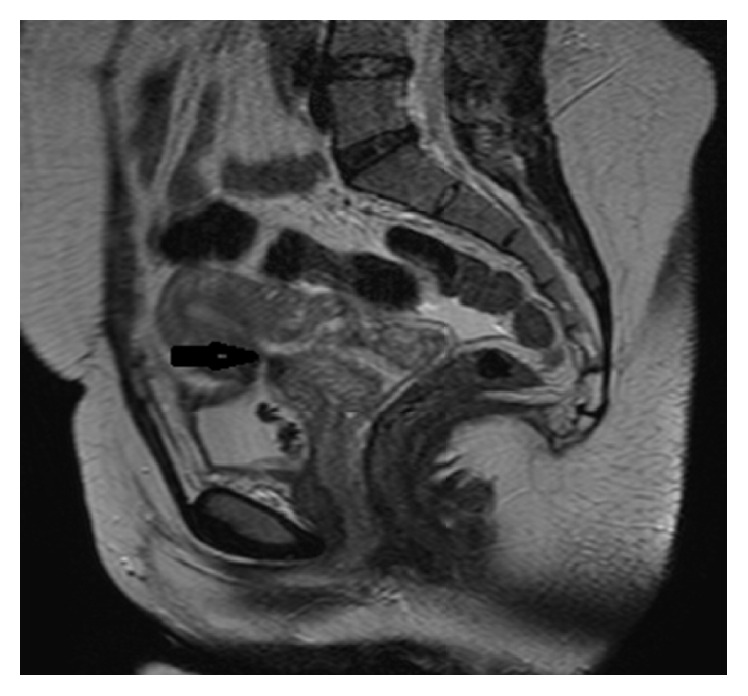
Fistula tract between the uterus anterior wall and bladder posterior wall in MRI.

A 28-year-old and 4-year married woman was admitted to the gynaecology outpatient clinic. She had two caesarean sections two years apart. The second caesarean section was performed due to placenta previa totalis and was uneventful. The main complaints of the patient were pelvic pain, haematuria, and intermittent vaginal discharge started a week after caesarean delivery. The external genitalia were assessed as normal. The speculum examination showed minimal fluid in the posterior fornix after valsalva manoeuvre. Uterine and adnexial movements were painful by bimanual examination. Transvaginal ultrasound was within normal limits. In the pelvic magnetic resonance imaging (MRI), a fistula tract was observed between anterior uterine wall which was at the same segment of the caesarean section and the posterior bladder wall. The filling of the liquid to the uterine cavity and vagina from the fistula tract was observed in the images taken after filling the bladder retrogradely with the liquid containing gadolinium (Figure 1).

**Figure 2:**
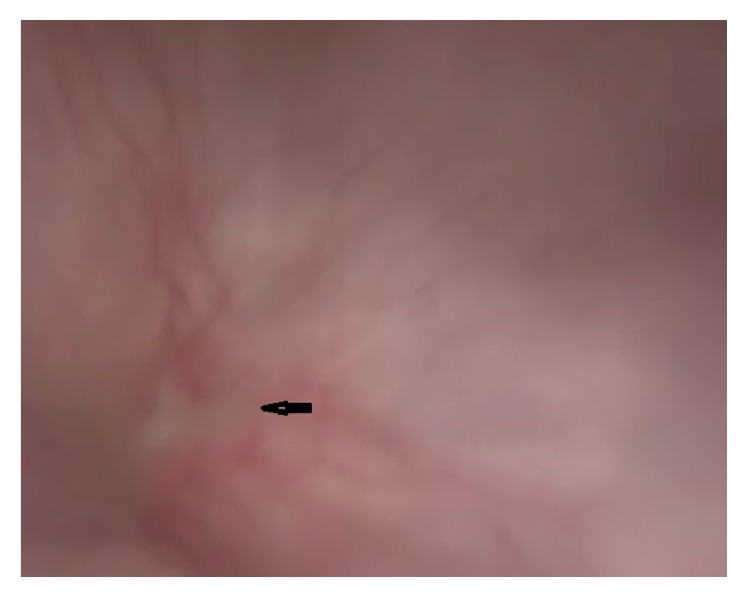
Fistula orifice in the bladder with cystoscopy.

**Figure 3:**
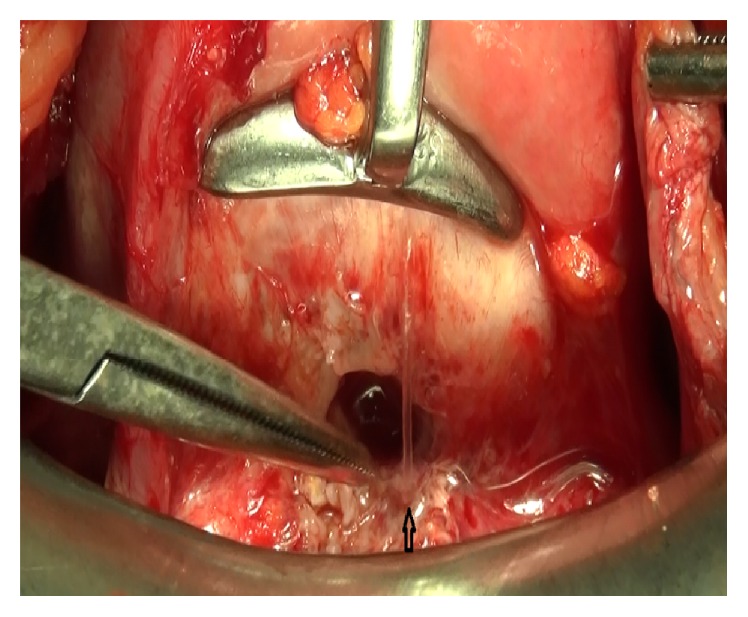
Operation image of the fistula tract in the uterus subsegment and bladder posterior wall.

**Figure 4:**
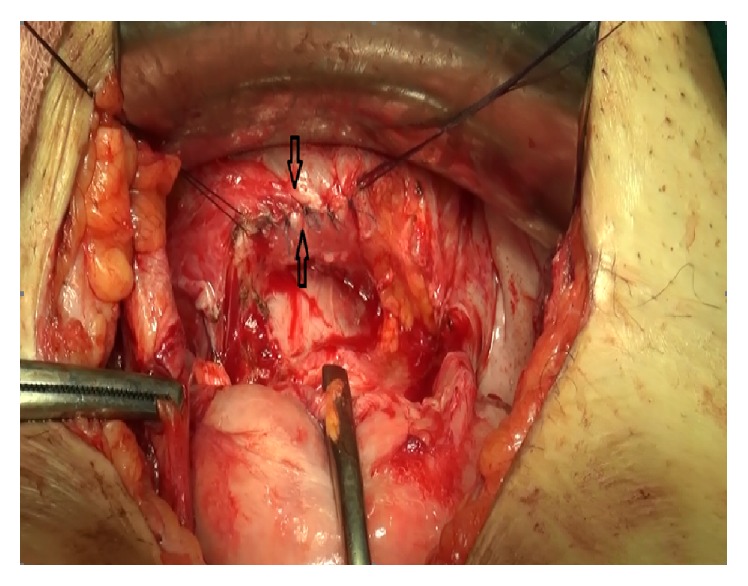
Dissection of the vesicouterine gap and repair of the bladder.

In the cystoscopic examination, a 1 cm fistula orifice was observed 2.5 cm above the trigone in the posterior bladder wall (Figure 2). Following the ascending bladder drainage and 2-week antibiotic therapy, the surgical treatment was planned for 3 months after the C/S. The fistula orifice was observed with cystoscopy before the surgery, and its position to the trigone and ureteral orifices was determined and the bladder mucosal integrity was observed. Transabdominal and transperitoneal approach was preferred. The uterovesical area was dissected with sharp dissection. The fistula tract between the subsegment section and bladder posterior wall was revealed (Figure 3). The fistula tract was excised. The defect on the posterior bladder wall was double-layer closed with absorbable sutures after tissue plans were being sufficiently mobilized (Figure 4). The water-tight closure of the bladder wall was checked with methylene blue dye test. An omental flap was placed between the uterus and the bladder by covering the uterus subsegment twice. After closing the abdomen, cystoscopy was repeated following placement of a drain in the uterovesical gap. Repaired fistula tract orifice, trigone, and ureter orifices were viewed. As no drainage was observed, the drain was pulled out on the fourth postoperative day. The bladder catheter was kept for 3 weeks. After bladder exercise, the bladder catheter was removed. She did not experience any complications including urinary incontinence and haematuria. After six months of follow-up, the patient had no complaints and was doing well and she is not planning a pregnancy for now.

## 3. Discussion

The World Health Organization (WHO) reports that 130.000 new urogenital fistula cases develop each year as a result of difficult deliveries [3]. However, it is estimated that the real incidence is higher as many women do not seek treatment in developing countries. While obstetric fistula is a phenomenon observed as a result of difficult deliveries and insufficient obstetric care in undeveloped and developing countries, UGFs generally occur as a result of gynecological interventions in developed countries [3].

VUFs, which are a rare type of urogenital fistulas, are also defined as Youssef's syndrome [2]. It was first described with the triad that was not accompanied by amenorrhoea, cyclic haematuria, and urinary incontinence following the subsequent caesarean delivery by Youssef in 1957. In previous years, it used to be regarded as a complication of assisted delivery applications like vacuum and forceps techniques. Today, 83–93% of VUFs are observed after caesarean delivery [4, 5]. Less frequently, this syndrome is observed following hysteroscopy as a complication of dilatation and curettage. Certain risk factors for VUFs are insufficient dissection of the bladder from the uterine subsegment, excessive intraoperative bleeding, the use of forceps and vacuum, placenta previa totalis, placental insertion abnormalities (acreata, inreata, and percerata), uterine rupture, previous caesarean section, and history of repeated abortions. Other less frequent causes are endometriosis, inflammatory bowel diseases, migration of the intrauterine devices, bladder tuberculosis, and congenital anomalies [5, 6]. In our case, the possible underlying mechanisms that caused VUF could be both caesarean delivery and excessive intraoperative bleeding due to placenta previa totalis.

VUFs may appear in different clinical presentations like amenorrhea, cyclic haematuria, pelvic pain, secondary infertility, and recurrent pregnancy losses, with or without involuntary urinary incontinence [4, 5].

While the constant urinary incontinence is imminent in the vesicovaginal fistula, this is subject to the level of the fistula tract in the VUFs. There may be menouria without urinary incontinence depending on the acting of a healthy cervix as a sphincter. While classic Youssef triad (the lack of amenorrhea, menouria, and urinary incontinence) is observed in the fistulas above the uterine isthmic level, menstruation is accompanied by urinary incontinence in the fistulas below it (incompetent cervix). That there is urinary incontinence from time to time in some cases is subject to the exceeding of the intrauterine pressure by the intravesical pressure and the drainage of the urine flow into the uterus from an incompetent cervix [1, 5–7].

There are three subtypes of Youssef's syndrome. Type 1 presented with menouria, Type 2 presented with urine flow into uterus and vagina, and Type 3 presented with normal menstrual cycles [8]. Our case was compatible with Type 2 Youssef's syndrome in terms of the findings. In our case, urinary incontinence was accompanied by other intermittent symptoms like pelvic pain, haematuria, and amenorrhea. However, cases with congenital development anomalies were found in the literature as well as those that were totally incidental [5, 9].

The diagnosis can be made using cystoscopy and radiological methods like ultrasonography, cystography, intravenous pyelography, cystoscopy, saline infusion sonohysterography, hysterosalpingography, helical computed tomography, and MRI [7, 10]. Radiologic screening methods also allow the surgeon to make the surgical treatment plan [10].

The double echogenic line between the uterus anterior wall and the posterior wall of the bladder in the ultrasound may suggest a VUF [11]. Different from vesicovaginal fistulas, cystography in VUF may be incompetent for the diagnosis as intrauterine pressure is higher than the intravesical pressure. Cystoscopy is important in terms of the determination and localization of the presence of a fistula and the determination of its position with the trigone. However, it is incompetent in determining the upper pole of the fistula tract. Despite the limitations of other diagnostic tests, diagnosis with MRI can be made with 100% accuracy. MRI also has the advantage of being noninvasive and does not contain ionizing radiation. MRI is regarded as the gold standard for the diagnosis and planning of the treatment [1, 3, 5, 7, 10].

In our case, the pelvic ultrasound was nondiagnostic. The diagnosis and the surgical treatment plan were made upon cystoscopy and MRI findings. The definitive treatment in VUF is surgery, besides conservative and expectant treatment approaches. Surgical treatment may be carried out via vaginal, transvesical, transperitoneal, laparoscopic, and robotic route [12, 13].

The expectant treatment method can be preferred in small fistulas. In this approach, the bladder is catheterized for 4–8 weeks, and amenorrhea is provided with oral contraceptives, progesterone, or gonadotropin hormone analogues [1, 3, 8]. In selected cases, cystoscopic fulguration may serve as a conservative approach in the presence of a small fistula [14, 15].

Expectant or surgical treatment can be preferred in cases that are symptomatic in the early postpartum period. Surgery should be planned in the first 48 hours for the cases with intensive haematuria and postpartum pain [15]. However, successful results were also reported in the periods up to days 5 and 17 [16]. Late period surgery should be planned for about 2-3 months after surgery. This period is necessary for the healing of inflammation and uterine involution.

We performed the surgical treatment with suprapubic, transabdominal, and transperitoneal approach in the postpartum third month. The bladder was catheterized for three weeks. Laparoscopic and robotic surgery approaches have similar success rates to open surgery in the surgical treatment of VUF. Minimally invasive techniques are superior to open surgery in terms of postoperative patient comfort and returning home early [16–20].

## 4. Conclusion

Vesicouterine fistulas known as Youssef's syndrome among genitourinary fistulas are rare fistulas. The number of increasing caesarean deliveries is the main cause of VUFs. As this syndrome may present in different clinical scenarios, suspicion, awareness, and further examination are important factors in the diagnosis. Cystoscopy is basic for diagnosis and exclusion of other possible lesions where MRI is basic for both the diagnosis and the planning of the treatment method. Laparoscopic and robotic surgeries should be preferred in the treatment of VUFs according to the surgeon's experience.
